# Declining Diversity in Abandoned Grasslands of the Carpathian Mountains: Do Dominant Species Matter?

**DOI:** 10.1371/journal.pone.0073533

**Published:** 2013-08-27

**Authors:** Anna Mária Csergő, László Demeter, Roy Turkington

**Affiliations:** 1 Department of Horticulture, Sapientia University, Târgu-Mureş, Romania; 2 Department of Environmental Engineering, Sapientia University, Miercurea-Ciuc, Romania; 3 Department of Botany and Biodiversity Research Centre, University of British Columbia, Vancouver, British Columbia, Canada; University of Alberta, Canada

## Abstract

Traditional haymaking has created exceptionally high levels of plant species diversity in semi-natural grasslands of the Carpathian Mountains (Romania), the maintenance of which is jeopardized by recent abandonment and subsequent vegetation succession. We tested the hypothesis that the different life history strategies of dominant grasses cause different patterns of diversity loss after abandonment of traditional haymaking in two types of meadow. Although diversity loss rate was not significantly different, the mechanism of loss depended on the life history of dominant species. In meadows co-dominated by competitive stress-tolerant ruderals, diversity loss occurred following the suppression of dominant grasses by tall forbs, whereas in meadows dominated by a stress-tolerant competitor, diversity loss resulted from increased abundance and biomass of the dominant grass. We conclude that management for species conservation in abandoned grasslands should manipulate the functional turnover in communities where the dominant species is a weaker competitor, and abundance and biomass of dominant species in communities where the dominant species is the stronger competitor.

## Introduction

Traditional landscapes are currently becoming a focus of biodiversity conservation efforts [1]. Among them, anthropogenic temperate grasslands attain global plant species maxima at scales ≤50 m^2^ [2]. Such high levels of diversity evolved during extended periods of traditional small-scale farming practices, but have been lost over the last century in most of Europe because of major socio-economic changes and unsustainable land use policies and practices. In the Carpathian Mountains of Eastern Europe, abandonment of traditional land use is rather recent, and abandoned grasslands are only now reverting to forests after a long period of arrested succession [3,4]. Much research effort is being focused on understanding the reasons for the high vascular plant diversity in traditionally managed meadows and the major species loss following abandonment [5]. Studies in various habitat types have shown that the identity of dominant species is critical to species diversity [6], compositional stability [7] and ecosystem functions [8]. The identity of dominant species may affect species establishment [6], species loss [9] and compensation for loss [10]. Moreover, the relative dominance of a single species may have higher impact on ecosystem functions than the community diversity itself (“mass ratio” versus “species diversity” hypotheses [8,11]). In spite of our growing knowledge of these processes, the effects of dominant species on diversity loss from these extremely diverse, now abandoned meadows, has received little study [12].

In empirical studies it is difficult to separate the effects of dominant species on diversity from that of external conditions, because dominance itself is a reflection of the environment and disturbance events [13,14]. Consequently, when subjected to similar disturbance regimes such as mowing, the diversity of two mature plant communities should diverge depending on the identity and abundance of the dominant species [12] and abiotic conditions [15] (but see 16,17 for the importance of regional species pools and land use history).

It is widely acknowledged that mowing increases levels of plant species diversity [5] by altering the balance of competition-colonization processes [13,14,18], niche overlap [19] and abiotic conditions [20]. Nevertheless, very little research has focused on the differences in species diversity induced in mowed grasslands by functionally different dominant species [21]. Conversely, cessation of mowing reverses the processes that cause high diversity and leads to species decline [22]. Usually, it is the gradually decreasing evenness of species in communities that increases the extinction risk [23]. Nonetheless, if the dominant species of two mature communities belong to different functional groups, unequal shifts in dominance and species loss rate can be expected.

Here we report the results of a study carried out in two types of hay meadow in the Carpathian Mountains. One is co-dominated by either *Festuca* *rubra* or *F* *. nigrescens* with *Agrostis* *capillaris* (Fescue meadows) and the other is dominated by *Brachypodium* *pinnatum* (Tor Grass meadows). These species are functionally different because *Festuca* spp. and *A* *. capillaris* develop small tussocks and thin stolons, whereas *Brachypodium* *pinnatum* develops only extravaginal tillers and strong belowground stolons [24]. According to Grime’s [13,24] classification of established life history strategies, *Festuca* spp. and *A* *. capillaris* are competitive stress-tolerant ruderals (CSR), whereas *Brachypodium* *pinnatum* is a stress-tolerant competitor (CS). Therefore we tested the following hypotheses: **1**. Species diversity will decline in abandoned meadows regardless of meadow type, because the species-rich state had been previously created and maintained by mowing. **2**. The abundance of dominant grasses will increase less (or will decrease) in Fescue- than Tor Grass meadows following abandonment because *Festuca* spp. and *Agrostis* *capillaris* are weaker competitors than *Brachypodium* *pinnatum* . **3**. Because *Festuca* spp. and *Agrostis* *capillaris* are weaker competitors, diversity loss following abandonment will be significantly slower in Fescue- than Tor Grass meadows. As a secondary objective, we tested whether abiotic conditions will lead to a higher variability of diversity patterns that would be generated by abandonment and meadow type alone.

The life history strategy of dominant species was an important driver of species depletion in the studied abandoned hay meadows. This confirms the deterministic nature of species loss and carries implications for managers of traditional agricultural landscapes.

## Methods

### Ethics statement

Our study was carried out entirely on private lands. We sampled the vegetation on several different farms, where landowners of these traditionally managed grasslands were at ease with giving permission to work. On a few occasions we did encounter red listed species, but there is no formal mechanism in Romania for getting permission to study such species outside protected areas. However, our study was purely observational and nondestructive, so when we did encounter rare species they were merely recorded along with all other species in plots. In addition, in all sites at some time during the growing season the farmers used to mow these grasslands (along with the rare species), which is a part of the long-term management practices that have occurred in these areas.

### Site description

**Figure 1:**
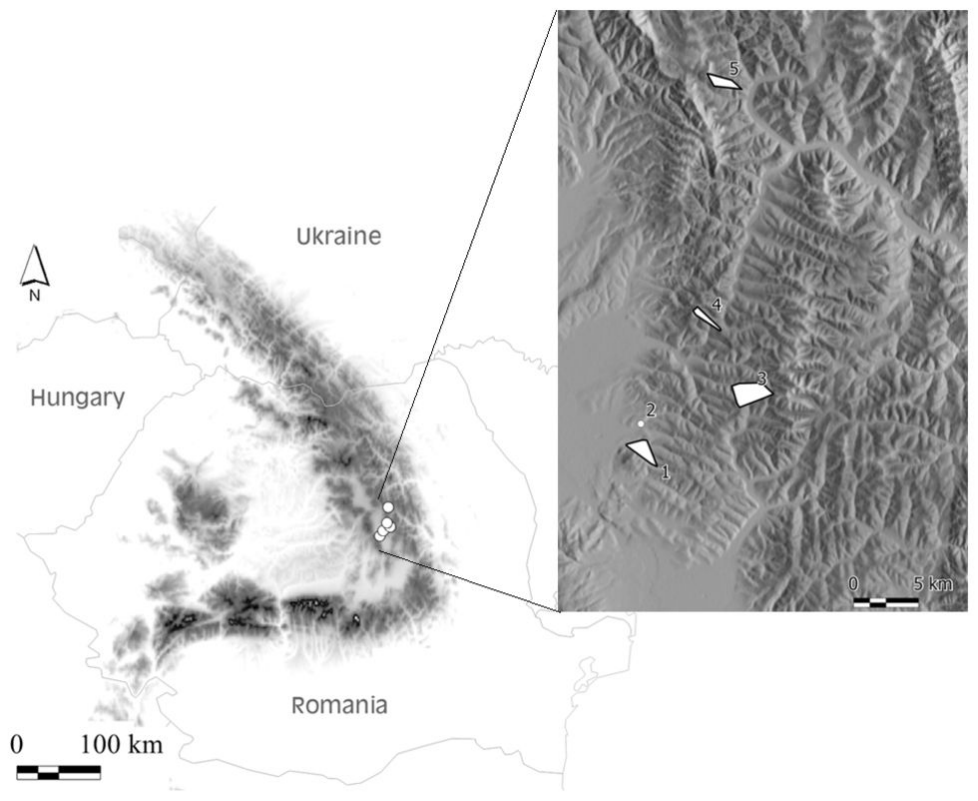
Map of the research area in the Carpathian Mountains (46^°^41’ N, 25^°^94’ E). The left panel indicates the geographic position of the five sampling sites within the Carpathian Mountains in Romania, Eastern Europe. The right panel shows the approximate area sampled at each site. Legend: 1 – Somlyó/ Șumuleu (SOM), 2 – Csomortán/ Șoimeni (CSOM), 3 – Kolos/Kolos (KOL), 4 – Pogányhavas/Muntele Păgân (POG), 5 – Jávárdi/Iavardi (JÁV).

The study was conducted in the Ciucului Mountains of the Southeastern Carpathians (46^°^41’ N, 25^°^94’ E), in the historic province of Transylvania in Romania, at altitudes between 749–1328 m (Figure 1). The climate is boreal-mountainous and average total annual precipitation ranges between 580–1200 mm. Hay meadows are a dominant landscape feature, and there is high variation of land use and topoclimatic conditions in the area. Meadows are traditionally mown by hand using a scythe. Sheep may temporarily graze the meadows at least once a year (mostly in autumn). The steep foothills were probably terraced in the Middle Ages as in other areas of Romania [25], and the process continued in the 18^th^ century. Terraces were ploughed until the second half of the 20^th^ century, when they were abandoned for hay making. Since the breakdown of socialist land use policies in the late 1980s, large grassland areas including terrace slopes have been abandoned, and large parts of the landscape currently experience secondary succession [3]. The time since abandonment in the studied plots was between one and five years based on interviews with land-owners and on tree ring counts.

### Dominant species

*Festuca* spp. and *A* *. capillaris* are much weaker competitors than *B* *. pinnatum* , because *B* *. pinnatum* has a competitive index (calculated from differences in height, growth form and yearly accumulation of litter) of 7-7.5, while the other three species each have a competitive index of 3.5 [24,26]. *Festuca* spp. and *A* *. capillaris* have wider ecological tolerance than *B* *. pinnatum* , but *B* *. pinnatum* is more successful than *Festuca* spp. in drier conditions [27]. Both Fescue- and Tor Grass meadows are widespread in hill and mountain areas of the Carpathian Basin, and develop mature equilibrial structure maintained by traditional management. In the Carpathian Mountains, Tor Grass meadows are less common at high elevations than on foothills and naturally occur at forest-grassland ecotones and following clear-cutting of forest. These Tor Grass meadows are gradually replaced by Fescue meadows under mowing. When managed, both meadow types have high levels of diversity and consequently are protected in Europe [28]. The nomenclature of species follows [29].

### Sampling procedure

Because abandonment was not studied experimentally, we conducted interviews with land-owners and a posteriori statistical analyses to detect site-specific reasons for abandonment. Generally, abandonment at the studied sites appeared to be driven by random factors which perhaps related to farmer’s individual choices rather than to distance from the villages, hay quality or site ecological conditions (unpublished results).

Five sites were chosen to account for the high topographic variability of the study area: two lower elevation (749-818 m) sites, (Somlyó/Șumuleu (SOM), Csomortán/Șoimeni (CSOM)), and three at higher (942-1328 m) elevations, Kolos/Kolos (KOL), Jávárdi/Iavardi (JÁV) and Pogányhavas/Muntele Păgân (POG) (Figure 1). We wished to sample combinations of two types of land use (mown and abandoned) and two dominant species (Fescue meadows and Tor Grass meadows), but because all four combinations did not occur at every site, four sites were chosen to represent each dominant grass: SOM, KOL, JÁV and POG for Fescue meadows, and SOM, KOL, JÁV, CSOM for Tor Grass meadows. Each site had both mown and abandoned meadows, except JÁV where all Tor Grass meadows were abandoned. Low elevation Tor Grass meadows were situated exclusively on terraced foothills, where terraces were mown and terrace slopes were previously mown, now abandoned. To account for environmental heterogeneity within each meadow type, the available combinations were subsampled with 4 to 13, randomly placed 1 m^2^ subplots, totaling 128 subplots. The percentage cover of each vascular plant species and of total vegetation was estimated visually in each subplot. Geographic coordinates, elevation, slope angle and aspect were extracted from GPS records and topographic maps (Vegetation data are provided in Table S1).

### Data analysis

Species diversity was measured as species richness (number of species / 1m^2^ subplot) and species evenness (*E*_*var*_ [30]). Potential direct incident solar radiation (*PDIR*) and heat load (HL) were computed from slope angle, aspect and geographical coordinates, following [31]. A two-way Analysis of Variance (ANOVA) was used to obtain mean differences in diversity estimates between the four combinations of land use and dominant species (i.e., meadow types) (procglm in SAS 9.2 [32]). The dataset was slightly unbalanced because of missing mown Tor Grass meadows in one locality (JÁV) and because two sites had either only Fescue (POG) or Tor Grass (CSOM) meadows, and consequently in this first exercise we followed a complete randomized design in which sites were used as replicates instead of blocks. Land use and dominant species were used as fixed effects, and replicates were introduced as random effects to control for variability among sites. Residuals were checked for normality (Kolmogorov-Smirnov test) and homoscedasticity (Bartlett test). Subsequent pairwise comparisons were performed using Bonferroni-corrected t-tests. The model was compared with an Analysis of Covariance (ANCOVA) to factor out existing differences due to abiotic site conditions. In addition to elevation, one of the following topoclimatic parameters: slope, potential direct incident solar radiation (*PDIR*) or heat load (HL) was chosen based on the strongest linear relationship with the dependent variables. Because *HL* was highly correlated with *PDIR* (Pearson’s ρ=0.820, p<0.001), this variable was abandoned in subsequent analyses. The relationships between species richness and species evenness were analyzed for both meadow types using Pearson correlations.

To evaluate whether abandonment differentially affected abundance of the two dominant species, a one-way ANOVA was conducted on each meadow type separately, following a randomized complete block design. First, percentage cover of dominant species was standardized by total vegetation cover within a plot. The relative cover resulted was used as a dependent variable, land use as fixed effect, and four (Fescue) and three (Tor Grass) sites were used as blocks. Residuals were checked for normality and homoscedasticity and transformation of the percentage cover values was not required. For Fescue meadows, the abundance of the codominant *A* *. capillaris* was added to the abundance of *Festuca* spp. to compute the final cover values of dominant species. Subsequent pairwise comparisons were performed using Bonferroni-corrected t-tests. To factor out confounding effects of the environment, the abiotic parameters were included as covariates in additional ANCOVAs, similarly to the first test.

To investigate how abundance of the two species impacted diversity relative to abiotic factors, partial regressions were produced separately for each meadow type (partial statement, multiple linear regression models, SAS; *N*_Fescue_=67, *N*_Tor Grass_=52). The partial regressions were built using residuals of diversity estimates and of dominant species relative cover. Residuals of diversity estimates were saved from a full multiple linear regression model where the relative cover was omitted and explanatory variables were elevation, *PDIR* and slope. Residuals of dominant relative cover were saved from a second full model where the dominant relative cover was regressed on the remaining regressors.

## Results

### Effect of abandonment and meadow type on diversity

**Figure 2:**
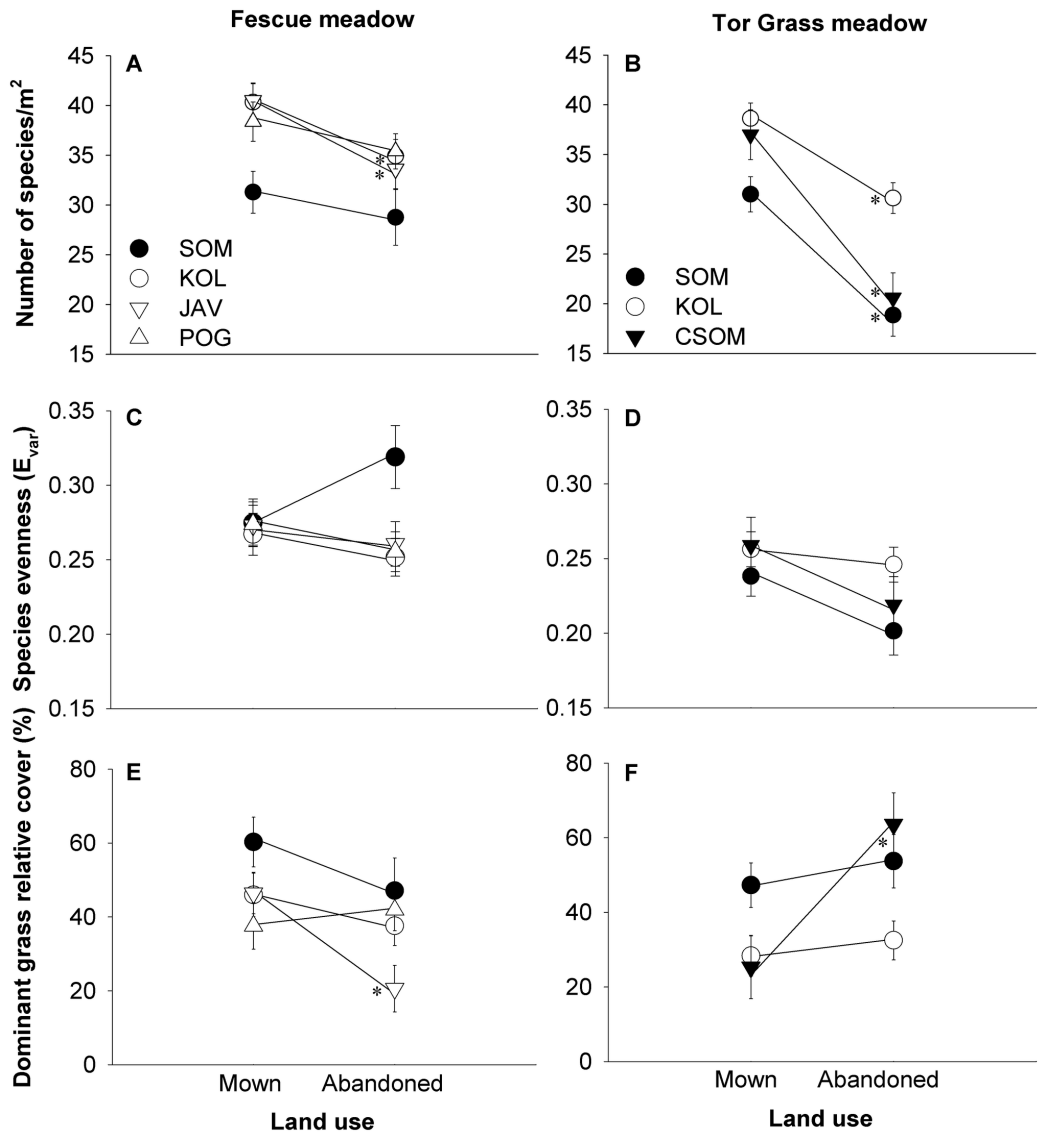
Mean (± 1SE) species diversity and dominant grass relative cover in combinations of meadow types and land use regimes. Diversity is measured as species richness (top panels) and species evenness (middle panels). Significant differences (ANOVA, post-hoc Bonferroni-corrected pairwise t tests; p<0.05) between mown and abandoned meadows are marked with an asterisk. Low elevation sites are shown in black. Abbreviations: SOM – Somlyó/ Șumuleu, CSOM – Csomortán/ Șoimeni, KOL – Kolos/Kolos, POG – Pogányhavas/Muntele Păgân, JÁV – Jávárdi/Iavardi.

There was a significantly lower species richness in abandoned than mown meadows (29.6±1.9 vs. 36.6±1.9 species, *p*=0.023) and a marginally significant higher species richness in Fescue- than Tor Grass meadows (35.4±1.8 vs. 30.8±1.9 species [means ±1*SE*], *p*=0.109) (ANOVA, F_[14,113,0.05]_=9.53, *p*_model_<0.001). Elevation had a significant effect on species richness, with 36.2±0.7 species in high elevation-, and 28.0±1.3 species in low-elevation meadows (means ±1*SE*; *p*=0.051, F_[16,111,0.05]_=8.73, p<0.001. There was a marginal interaction of *PDIR* with replicates (sites), with low elevation abandoned Tor Grass meadows (terrace slopes) having higher *PDIR* and lower richness (19.6±1.2) than their mowed Tor Grass counterparts on terraces (33.0±2.1) (means ±1*SE*; *p*=0.102, ANCOVA). Slope on its own had no significant effect on species richness (*p*<0.392) (ANCOVA, F_[15,112,0.05]_=9.19, *p*_model_<0.001). Due to a significant variation of species richness across sites (*p*<0.001) (Figure 2A,B), no significant land use × meadow type interaction was detected in either the ANOVA or ANCOVA model (*p*>0.270). Despite this non-significant result, species richness in abandoned versus mowed meadows was lower with an average of only 4.6 species/m^2^ (12.0%) in Fescue meadows, compared to 8.9 species/m^2^ (26.9%) in Tor Grass meadows (ANCOVA-corrected means).

Only meadow type had a significant effect on species evenness (*p*_meadow type_=0.021, *p*_land use_=0.445), with slightly higher values in Fescue- than Tor Grass meadows (0.27±0.007 vs. 0.24±0.008) (means ±1*SE*) (ANOVA, F_[14,113,0.05]_=2.36, *p*_model_ =0.007). There was no significant interaction of abiotic variables with either (i) site (*p*>0.270, ANCOVA) or (ii) species evenness (*p*>0.121) (ANCOVA, F_[16,111,0.05]_>2.18, *p*_model_<0.05). Due to a significant variation of evenness across sites (*p*<0.048) (Figure 2C,D), no land use × meadow type interaction was detected in either the ANOVA or ANCOVA model (*p*>0.467) (The SAS code and detailed results for this section are provided in Material S1).

The relationship of species richness to species evenness was positive and significant only in Tor Grass meadows (Pearson’s *r*=0.190, *p*=0.123 in Fescue-, and *r*=0.569, *p*<0.01 in Tor Grass meadows).

### Effect of abandonment on dominant species cover

The summed relative cover of *Festuca* spp. with *A* *. capillaris* , and of *B* *. pinnatum* was not significantly different in abandoned versus mown plots (36.8%±4.9 vs. 47.6%±4.5, *p*=0.207 and 49.9%±7.2 vs. 33.6%±6.9[means ±1*SE*], *p*=0.245 respectively) (ANOVA, *F*_Fescue[7, 59, 0.05]_=3.08, *p*_model_=0.008; *F*_Tor Grass[5, 46, 0.05]_=4.51, *p*_model_=0.002). The differences were dampened by land use × site interactions in both meadows (*p*_Fescue_=0.100, *p*_Tor Grass_=0.053) (Figure 2E,F). In Fescue meadows there was a combined interaction of elevation and slope with sites (p<0.087), high-elevation meadows having steeper slopes and lower dominant grass cover than lower elevation meadows (means ±1*SE* = 38.9±2.6 vs. 55.5±4.6). No significant interaction of either abiotic parameter with site existed in Tor Grass meadows (*p*>0.321). Neither elevation, *PDIR* or slope on their own had an effect on the relative cover of the dominant grass in either land use model (*p*>0.335) (ANCOVA *F*_Fescue[8, 58, 0.05]_<3.91, *p*_model_<0.05; *F*_Tor Grass[6, 45, 0.05]_<2.67, *p*_model_<0.05) (The SAS code and detailed results for this section are provided in Material S2).

The relative cover of dominant grasses averaged over mowed and abandoned meadows was similar in the two meadow types (mean ±1*SE*=41.6%±2.4 in Fescue-, and 39.5%±3.0 in Tor Grass meadows).

### Effect of dominant species cover on diversity

**Figure 3:**
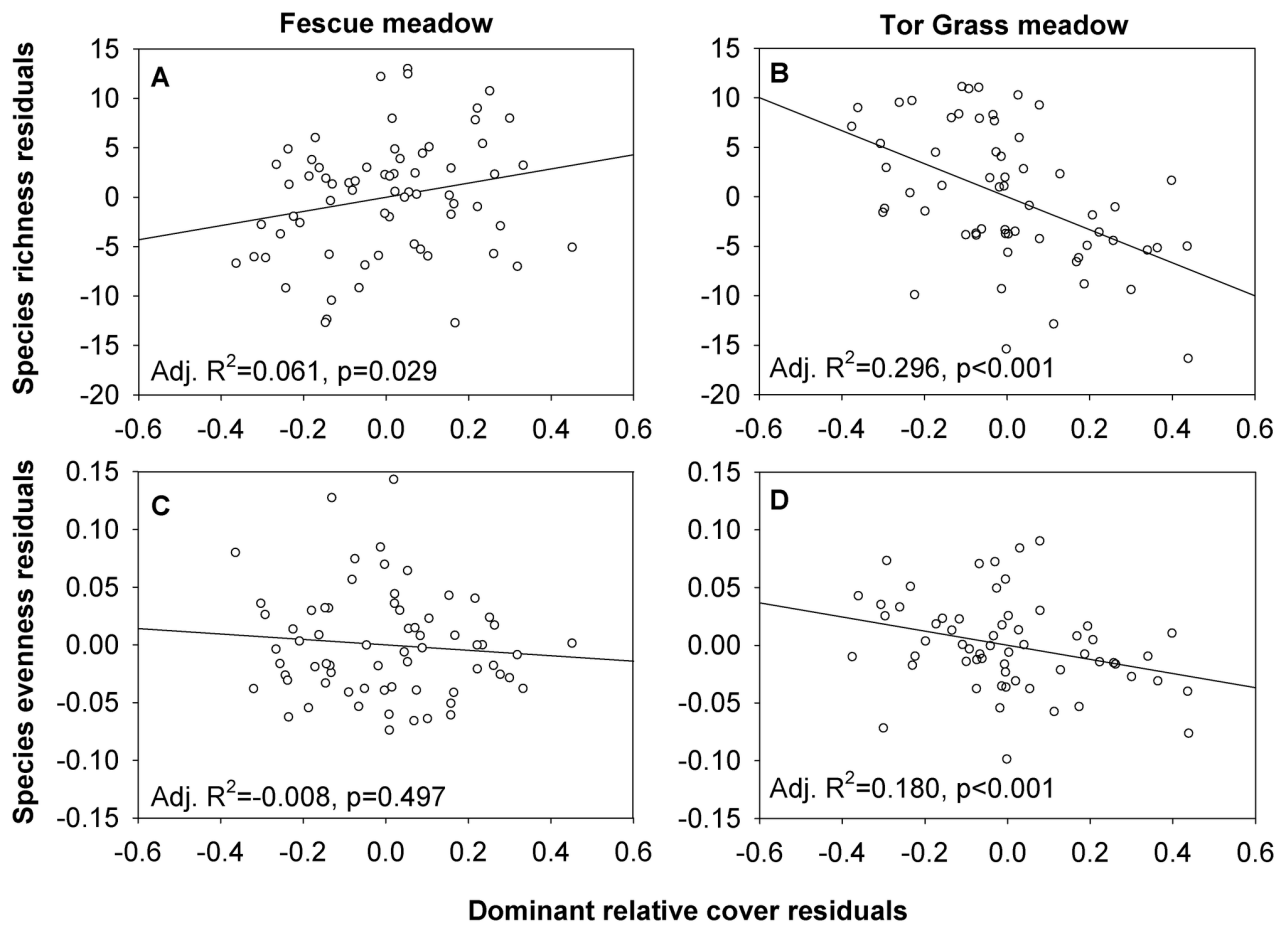
The relationship of dominant grass cover to species diversity. Partial regressions of the dominant grass relative cover and species richness (top panels) and species evenness (bottom panels) in two meadow types, after accounting for the effect of abiotic variables.

After accounting for the effect of environmental parameters (elevation, slope, *PDIR*), the relative cover of *Festuca* spp. with *A* *. capillaris* had a weak positive effect, and *B* *. pinnatum* relative cover had a stronger negative effect on species richness (Figure 3A,B). In Fescue meadows, dominant grass relative cover had no effect on species evenness (Figure 3C), whereas in Tor Grass meadows the relationship was negative and significant (Figure 3D).

## Discussion

The mountain hay meadows in the Carpathian Mountains are a highly diverse anthropogenic grassland ecosystem that is threatened by cessation of traditional management. Averaging 40.5 plant species/m^2^ at local (site) level and reaching 50 species/m^2^ (likely even more following additional surveys in spring), these hay meadows are among the most diverse semi-natural grasslands in Europe, which average 40.4 species/m^2^ and have up to 79 species/m^2^ [2 and references therein]. Long-term moderate disturbance (mowing and seasonal light grazing) has caused convergent and high levels of diversity in both Fescue and Tor Grass meadows. The cause of species depletion following abandonment is strongly dependent on the life history strategy of the dominant species, which confirms the deterministic nature of species loss [9]. In meadows co-dominated by the CSR strategists and weaker competitors, *Festuca* spp. and *A* *. capillaris* , diversity loss occurs because the dominant grasses are suppressed by forbs such as *Laserpitium* *latifolium* and *Trollius* *europaeus* . In meadows dominated by the CS strategist and stronger competitor *B* *. pinnatum* , diversity loss results from increased abundance and biomass of dominant species.

### Effect of abandonment on diversity

Our results support hypothesis 1 i.e, species diversity declined in abandoned meadows regardless of meadow type. Following only one to five years of abandonment, grassland diversity declined by 12 and 27% in Fescue- and Tor Grass meadows respectively, which is a worrying loss. Similar depletion rates were reported from other previously mown and abandoned grasslands (e.g. 21-57% loss in 10-20 years [33]). Although we expected that species loss would be driven by decreasing evenness as in most abandoned meadows [23], the significant driver of evenness patterns was the meadow type, and not abandonment. With a similar overall abundance of dominant grasses in the two meadows, a lower evenness in Tor Grass meadows may be explained by the fewer and less abundant species, which is an intrinsic feature of this plant community. The slight decrease of evenness following abandonment was caused by increasing abundance of *B* *. pinnatum* in Tor Grass meadows and increased cover of a few tall forb species in both meadows (e.g. *Laserpitium* *latifolium* , *Trollius* *europaeus* *,* *Salvia* *pratensis* *,* *Chrysanthemum* *corymbosum* *,* *Betonica* *officinalis* ), a pattern often reported in early stages of abandonment [34]. Species depletion from abandoned meadows is to be expected because mowing prevented the expression of competitive dominants and maintained the grasslands in a relatively stable species-rich state. With abandonment there is a reversion towards a species-poorer mixed temperate forest, a cycle often seen in periodically perturbed ecosystems [35]. Bushes of *Salix* *triandra* and saplings of *Populus* *tremula* and *Picea abies* were common in our abandoned meadows, and young poplar stands can dominate the meadows very quickly (pers. obs.). Nonetheless, over a short abandonment period a general decrease of diversity is not necessarily accompanied by a declining number of species having a high conservation value. In Fescue grasslands for example, a similar number of red-listed species were found under both management regimes, and some of the species were unique to abandoned meadows (unpublished results).

### Effect of abandonment on dominant species cover

Our results support hypothesis 2 i.e, the abundance of dominant grasses increase less or even decrease in meadows dominated by weaker competitors, because the weak competitors *Festuca* spp. and *A* *. capillaris* had lower cover, whereas the stronger competitor *B* *. pinnatum* had higher cover in abandoned compared to mowed meadows.

When mown, all studied species lose significant above- and belowground biomass [36,37]. However, *Festuca* spp. and *A* *. capillaris* are more resistant to defoliation [38], can develop compensatory growth responses to clipping [39] and disturbance can enhance their recruitment [40], making them good mowing indicators [41]. However, despite a high clonal mobility, *F* *. rubra* is sensitive to shading and the identity of its neighbors [42] and is disadvantaged when overgrown by a herbaceous canopy. Therefore, in abandoned meadows where light becomes limiting, *Festuca* spp. is vulnerable to displacement.

In contrast, *B* *. pinnatum* is larger than *Festuca* spp. and *A* *. capillaris* and consequently is more vulnerable to nitrogen and carbohydrate loss when mown. This is critical for regeneration of species with underground rhizomes and stolons after mowing because taller species are affected more than shorter species [21,39]. Indeed, Pons et al. [43] and Bobbink and Willems [44] showed that leaf nitrogen is concentrated in the upper part of a *B* *. pinnatum* canopy and nitrogen loss is the main cause of the species’ decline when mown. Following cessation of mowing, *B* *. pinnatum* increased cover probably as a response to release from mowing suppression, and due to its ability to regenerate from belowground structures [36]. However, there were no overall significant quantitative changes in abundance of either of the dominant grasses, presumably because of the short time since abandonment.

### Effect of dominant species on diversity

Hypothesis 3 is rejected because diversity loss following abandonment was not significantly lower in meadows dominated by the weaker competitors *Festuca* spp. and *A* *. capillaris* . Although the decline of species richness and evenness in Fescue meadows was, on average, less than in Tor Grass meadows following abandonment, differences were not significant because of high landscape-level variability of diversity patterns in both meadow types.

However, opposite effects of abandonment on dominant grass abundance triggered different mechanisms of species loss. In mowed Fescue meadows, reduced competition and lower niche overlap increased species richness while at the same time enhanced vegetative propagation and seedling recruitment of dominant species [19,40,42]. Following abandonment, increasing competition for light reversed the relative importance of these mechanisms and caused both species richness and dominant species cover to decline. Evenness did not decrease substantially because dominant grasses were overtopped by a few stronger competitive forbs (e.g. *Trollius* *europaeus* ). Such a rapid turnover of functional groups is specific to species-rich communities with even dominance distributions [45] and in our system seems to be dependent upon management regime (i.e., mowing or abandonment). Consequently, in Fescue meadows the effect of dominant grasses on species loss following abandonment appears to be very weak. Not surprisingly, species richness is independent of species evenness.

In contrast, abandoned Tor Grass meadows lost more species than Fescue meadows at all sites. The difference may partly be attributed to higher *B* *. pinnatum* cover, which concurs with current evidence on compositional instability of abandoned Tor Grass meadows [36]. The causes of the negative impact of *B* *. pinnatum* on diversity may include its high competitive superiority and ability to slow down species turnover [46,47]. However, the abundance of *B* *. pinnatum* generally increased very little with abandonment, and other factors such as copious litter accumulation (pers. obs.) might have had a stronger negative impact on diversity [46]. As expected, the lower species richness of Tor Grass meadows was related to reduced community evenness, similarly to other dominant species [23]. Consequently, abundance and biomass of *B* *. pinnatum* are critical to species loss from Tor Grass meadows following abandonment. Such fundamental differences among dominant species may be important to conservation managers, having the potential to predict the resistance or resilience of local communities to diversity loss following abandonment [45,48].

### Effect of environment on diversity

Abiotic conditions added high variability to the expected diversity patterns. Species richness, evenness and dominant species relative cover were all affected by significant interactions with sites. Elevation had positive effect on species richness with, for example, more endemic species added to the regional pool. The Eastern Carpathian Mountains is a region of high endemism [49] and most of these endemic species occur in high elevation grasslands. Likewise, climate-driven geographic distributions augmented diversity of other high elevation grasslands such as those in the Appalachian Mountains [50]. In addition, in high elevation Fescue meadows on steeper slopes, dominant grasses had lower cover and all other species higher cover than at low elevations. The effect of slope angle is probably confounding with the distribution of management regimes. Low elevation, less steep meadows are close to human settlements and have probably been subjected to a more intensive mowing regime, which is known to increase *Festuca* spp. abundance and decrease diversity [41]. Therefore, moderate management regimes of high elevation meadows are better alternatives of land use practices in terms of plant diversity [51].

High solar radiation in abandoned Tor Grass meadows on terrace slopes was related to decreased levels of diversity. This effect often occurs under low moisture conditions on steep slopes associated with high solar radiation [52]. In abandoned meadows subjected to drought conditions, subordinate species have first to pass through abiotic filters, and then compete with dominant species for nutrients or space [53], which strongly reduced diversity in our system. However, terrace slopes allowed rare stress tolerators with low competitive ability (e.g. *Aster* *amellus* *,* *Linum* *hirsutum* ) to persist within the abandoned grasslands. Consequently, despite producing lower local diversity, particular topographic conditions may increase the landscape-level species pool of hay meadows and are valuable for conservation [54].

## Conclusions

In abandoned hay meadows of the Carpathian Mountains, the life history strategy of the dominant species is an important driver of species loss. This result is critical for conservation and for land managers of traditional agricultural landscapes. In communities dominated by weak competitive grasses, maintaining high plant diversity is not achieved by manipulating dominant grass cover, but rather, by preventing species-poor assemblages of tall forbs from becoming dominant on the long run. In contrast, in communities dominated by competitive grasses, manipulation of dominant species’ abundance and biomass are both crucial to the maintenance of high diversity. Despite a significant loss of plant diversity, short-term abandonment episodes may not be entirely detrimental to the grassland ecosystems and benefits to rare plant preservation or invertebrate communities (as in [55]) should be more closely examined. In addition, high landscape-level heterogeneity may produce site-specific diversity patterns in both types of grasslands, which highlights the complexity of factors that modulate coexistence processes at a regional scale and the need to adjust the management practices accordingly.

## Supporting Information

Material S1 **SAS code and table of results for the ANOVA and ANCOVA models testing for the effect of abandonment and meadow type on diversity in the Carpathian Mountains.**
(DOCX)

Material S2 **SAS code and table of results for the ANOVA and ANCOVA models testing for the effect of abandonment on dominant species cover in two types of meadow in the Carpathian Mountains.**
(DOCX)

Table S1 **Vegetation data in 1 m^2^ subplots sampled in two different types of meadow under mowing and abandonment in the Carpathian Mountains.**
(XLS)
