# Supplementary material for: Declining Diversity in Abandoned Grasslands of the Carpathian Mountains: Do Dominant Species Matter?
Source: PLoS One. 2013 Aug 27;8(8):e73533. doi: 10.1371/journal.pone.0073533 (PMC3754964; doi:10.1371/journal.pone.0073533)
Supplement: Material S1 — SAS code and table of results for the ANOVA and ANCOVA models testing for the effect of abandonment and meadow type on diversity in the Carpathian Mountains. (DOCX) [file pone.0073533.s001.docx]

Abbreviations:

Y1=Species richness

Y2=Species evenness

A=Land use regime

B=Dominant species (i.e, Meadow type)

R=Site

X1=Altitude

X2= Potential Direct Incident Radiation

**1A)** SAS 9.2 code and table of the results for the Two-way ANOVA for testing mean differences in species richness estimates between the four combination of land use and dominant species.

*#MODEL: Two-way ANOVA, with A and B as fixed effects and R(A*B) as random effect*

*PROC IMPORT OUT=WORK.M1 DATAFILE="C:\AnnaMaria\abandonment.XLS" DBMS=EXCELCS REPLACE;*

*RANGE="Sheet1$";*

*SCANTEXT=YES;*

*USEDATE=YES;*

*SCANTIME=YES;*

*RUN;*

*OPTIONS LINESIZE=78 PAGESIZE=60;*

*PROC GLM DATA=M1;*

*CLASS A B R;*

*MODEL Y1=A B A*B R(A*B)/SS3;*

*LSMEANS R(A*B)/STDERR TDIFF;*

*TEST H=A B A*B E=R(A*B);*

*LSMEANS A B A*B/STDERR TDIFF E=R(A*B);*

*RUN;*

*OUTPUT OUT=RES1 PREDICTED=PREDICT RESIDUAL=RESID;*

*PROC UNIVARIATE DATA=RES1 PLOT NORMAL;*

*VAR RESID;*

*RUN;*

*PROC ANOVA DATA=RES1;*

*CLASS A B R;*

*MODEL RESID = A*B;*

*MEANS A*B/HOVTEST=BARTLETT;*

*RUN;*

Two-way ANOVA results for species richness

| **Source** | **df** | **Mean square** | **F** | **p** |
| --- | --- | --- | --- | --- |
| Land use | 1 | 1356.912 | 6.91 | 0.023 |
| Dominant species | 1 | 606.978 | 3.09 | 0.106 |
| Land use × dominant species | 1 | 177.390 | 0.90 | 0.362 |
| Site (land use × dominant species) | 11 | 196.305 | 6.26 | <0.001 |
| Sampling error | 113 | 31.358 | - | - |

**1B)** SAS 9.2 code and table of the results for the ANCOVA for testing mean differences in species richness estimates between the four combination of land use and dominant species, having elevation and Potential Direct Incident Radiation as a covariates.

*# MODEL: ANCOVA, with A and B as fixed effects and R(A*B) as random effect, an X1 and X2 as covariates:*

*PROC IMPORT OUT=WORK.M1 DATAFILE="C:\AnnaMaria\abandonment.XLS" DBMS=EXCELCS REPLACE;*

*RANGE="Sheet1$";*

*SCANTEXT=YES;*

*USEDATE=YES;*

*SCANTIME=YES;*

*RUN;*

*OPTIONS LINESIZE=78 PAGESIZE=60;*

*# testing the interactions with X1 and X2*

*PROC GLM DATA=M1;*

*CLASS A B R;*

*MODEL Y1= X1 X2 A B A*B R(A*B) X1*R(A*B) X2*R(A*B)/SS3;*

*RUN;*

*# the interaction X2*R(A*B) was significant at the set level (p=0.1018), and therefore we repeated the analysis with X1 only*

*PROC GLM DATA=M1;*

*CLASS A B R;*

*MODEL Y1= X1 A B A*B R(A*B) X1*R(A*B)/SS3;*

*RUN;*

*PROC GLM DATA=M1;*

*CLASS A B R;*

*MODEL Y1= X1 A B A*B R(A*B)/SS3;*

*TEST H=A B A*B E=R(A*B);*

*LSMEANS R(A*B)/STDERR TDIFF;*

*LSMEANS A B A*B/STDERR TDIFF E=R(A*B);*

*RUN;*

*OUTPUT OUT=RES1 PREDICTED=PREDICT RESIDUAL=RESID;*

*PROC UNIVARIATE DATA=RES1 PLOT NORMAL;*

*VAR RESID;*

*RUN;*

*PROC ANOVA DATA=RES1;*

*CLASS A B R;*

*MODEL RESID = A*B;*

*MEANS A*B/HOVTEST=BARTLETT;*

*RUN;*

ANCOVA result for species richness

| **Source** | **df** | **Mean square** | **F** | **p** |
| --- | --- | --- | --- | --- |
| Elevation | 1 | 120.256 | 3.90 | 0.051 |
| Potential Direct Incident Radiation | 1 | 44.159 | 1.43 | 0.234 |
| Land use | 1 | 1121.318 | 12.06 | 0.005 |
| Dominant species | 1 | 533.413 | 5.74 | 0.036 |
| Land use × dominant species | 1 | 125.523 | 1.35 | 0.270 |
| Site (land use × dominant species) | 11 | 92.976 | 3.02 | 0.002 |
| Sampling error | 111 | 30.822 | - | - |

**2A)** SAS code and table of the results for the Two-way ANOVA for testing mean differences in species evenness estimates between the four combination of land use and dominant species.

*# MODEL: Two-way ANOVA, with A and B as fixed effects and R(A*B) as random effect*

*PROC IMPORT OUT=WORK.M1 DATAFILE="C:\AnnaMaria\abandonment.XLS" DBMS=EXCELCS REPLACE;*

*RANGE="Sheet1$";*

*SCANTEXT=YES;*

*USEDATE=YES;*

*SCANTIME=YES;*

*RUN;*

*OPTIONS LINESIZE=78 PAGESIZE=60;*

*PROC GLM DATA=M1;*

*CLASS A B R;*

*MODEL Y2=A B A*B R(A*B)/SS3;*

*LSMEANS R(A*B)/STDERR TDIFF;*

*TEST H=A B A*B E=R(A*B);*

*LSMEANS A B A*B/STDERR TDIFF E=R(A*B);*

*RUN;*

*OUTPUT OUT=RES1 PREDICTED=PREDICT RESIDUAL=RESID;*

*PROC UNIVARIATE DATA=RES1 PLOT NORMAL;*

*VAR RESID;*

*RUN;*

*PROC ANOVA DATA=RES1;*

*CLASS A B R;*

*MODEL RESID = A*B;*

*MEANS A*B/HOVTEST=BARTLETT;*

*RUN;*

Two-way ANOVA results for species evenness

| **Source** | **df** | **Mean square** | **F** | **p** |
| --- | --- | --- | --- | --- |
| Land use | 1 | 0.002 | 0.63 | 0.445 |
| Dominant species | 1 | 0.025 | 7.32 | 0.021 |
| Land use × dominant species | 1 | 0.002 | 0.57 | 0.467 |
| Site (land use × dominant species) | 11 | 0.003 | 1.89 | 0.048 |
| Sampling error | 113 | 0.002 | - | - |

**2B)** SAS code and table of the results for the Two-way ANCOVA for testing mean differences in species richness estimates between the four combination of land use and dominant species, having elevation and Potential Direct Indirect Radiation as a covariates.

*# MODEL: ANCOVA, with A and B as fixed effects and R(A*B) as random effect, an X1 and X2 as covariates*

*PROC IMPORT OUT=WORK.M1 DATAFILE="C:\AnnaMaria\abandonment.XLS" DBMS=EXCELCS REPLACE;*

*RANGE="Sheet1$";*

*SCANTEXT=YES;*

*USEDATE=YES;*

*SCANTIME=YES;*

*RUN;*

*OPTIONS LINESIZE=78 PAGESIZE=60;*

*# testing the interaction with X1 and X2*

*PROC GLM DATA=M1;*

*CLASS A B R;*

*MODEL Y2= X1 X2 A B A*B R(A*B) X1*R(A*B) X2*R(A*B)/SS3;*

*RUN;*

*#there was no significant interaction with either covariates*

*PROC GLM DATA=M1;*

*CLASS A B R;*

*MODEL Y2= X1 X2 A B A*B R(A*B)/SS3;*

*TEST H=A B A*B E=R(A*B);*

*LSMEANS R(A*B)/STDERR TDIFF;*

*LSMEANS A B A*B/STDERR TDIFF E=R(A*B);*

*RUN;*

*OUTPUT OUT=RES1 PREDICTED=PREDICT RESIDUAL=RESID;*

*PROC UNIVARIATE DATA=RES1 PLOT NORMAL;*

*VAR RESID;*

*RUN;*

*PROC ANOVA DATA=RES1;*

*CLASS A B R;*

*MODEL RESID = A*B;*

*MEANS A*B/HOVTEST=BARTLETT;*

*RUN;*

ANCOVA final result for species evenness

| **Source** | **df** | **Mean square** | **F** | **p** |
| --- | --- | --- | --- | --- |
| Elevation | 1 | 0.004 | 2.00 | 0.160 |
| Potential Direct Incident Radiation | 1 | 0.001 | 0.58 | 0.448 |
| Land use | 1 | 0.001 | 0.34 | 0.570 |
| Dominant species | 1 | 0.019 | 5.17 | 0.044 |
| Land use × dominant species | 1 | 0.001 | 0.28 | 0.605 |
| Site (land use × dominant species) | 11 | 0.004 | 2.06 | 0.029 |
| Sampling error | 111 | 0.008 | - | - |
