# Supplementary material for: Declining Diversity in Abandoned Grasslands of the Carpathian Mountains: Do Dominant Species Matter?
Source: PLoS One. 2013 Aug 27;8(8):e73533. doi: 10.1371/journal.pone.0073533 (PMC3754964; doi:10.1371/journal.pone.0073533)
Supplement: Material S2 — SAS code and table of results for the ANOVA and ANCOVA models testing for the effect of abandonment on dominant species cover in two types of meadow in the Carpathian Mountains. (DOCX) [file pone.0073533.s002.docx]

**1A)** SAS 9.2 code and table of the results for the One-way ANOVA for testing mean differences in *Festuca* spp. cover between different types of land use regimes

Abbreviations

Y3=*Festuca* spp. relative cover

A=Land use regime

R=Site

X1=Elevation

X2c=Slope

*#MODEL: Randomized complete block design, with A as factor and R as blocks;*

*PROC GLM DATA=M2;*

*CLASS A R;*

*MODEL Y3=A R A*R/SS3;*

*TEST H=A R E=A*R;*

*LSMEANS A*R/STDERR TDIFF;*

*LSMEANS A R/STDERR TDIFF E=A*R;*

*RUN;*

*OUTPUT OUT=RES1 PREDICTED=PREDICT RESIDUAL=RESID;*

*PROC UNIVARIATE DATA=RES1 PLOT NORMAL;*

*VAR RESID;*

*RUN;*

*PROC ANOVA DATA=RES1;*

*CLASS A R;*

*MODEL RESID = A*R;*

*MEANS A*R/HOVTEST=BARTLETT;*

*RUN;*

ANOVA results for *Festuca* spp. cover

| **Source** | **df** | **Mean square** | **F** | **p** |
| --- | --- | --- | --- | --- |
| Land use | 1 | 0.178 | 2.57 | 0.207 |
| Site | 3 | 0.089 | 1.29 | 0.420 |
| Land use × Site | 3 | 0.069 | 2.18 | 0.100 |
| Sampling error | 59 | 0.032 | - | - |

**1B)** SAS 9.2 code and table of the results for the ANCOVA for testing mean differences in *Festuca* spp. cover between different types of land use regimes, having Elevation and Slope as a covariates.

*#MODEL: Randomized complete block design, with A as factor and R as blocks, and X1 and X2c as covariates;*

*# testing the interactions with X1 and X2c*

PROC GLM DATA=M2;

CLASS A R;

MODEL Y3= X1 X2c A R A*R X1*A*R X2c*A*R/solution SS3;

RUN;

ANCOVA result showing the interactions of Elevation and Slope with Land use × Site

| **Source** | **df** | **Mean square** | **F** | **p** |
| --- | --- | --- | --- | --- |
| Elevation | 1 | 0.083 | 3.41 | 0.072 |
| Slope | 1 | 0.114 | 4.73 | 0.035 |
| Land use | 1 | 0.093 | 3.85 | 0.056 |
| Site | 3 | 0.155 | 6.42 | 0.001 |
| Land use × Site | 3 | 0.039 | 1.61 | 0.202 |
| Elevation × Land use × Site | 7 | 0.083 | 3.43 | 0.005 |
| Slope × Land use × Site | 7 | 0.047 | 1.94 | 0.087 |
| Sampling error | 43 | 0.024 | - | - |

**2A)** SAS 9.2 code and table of the results for the One-way ANOVA for testing mean differences in *Brachypodium pinnatum* cover between different types of land use regimes

Abbreviations

Y3= *Brachypodium pinnatum* relative cover

A=Land use regime

R=Site

X1=Elevation

X2c=Slope

*#MODEL: Randomized complete block design, with A as factor and R as blocks;*

*PROC GLM DATA=M2;*

*CLASS A R;*

*MODEL Y3=A R A*R/SS3;*

*TEST H=A R E=A*R;*

*LSMEANS A*R/STDERR TDIFF;*

*LSMEANS A R/STDERR TDIFF E=A*R;*

*RUN;*

*OUTPUT OUT=RES1 PREDICTED=PREDICT RESIDUAL=RESID;*

*PROC UNIVARIATE DATA=RES1 PLOT NORMAL;*

*VAR RESID;*

*RUN;*

*PROC ANOVA DATA=RES1;*

*CLASS A R;*

*MODEL RESID = A*R;*

*MEANS A*R/HOVTEST=BARTLETT;*

*RUN;*

ANOVA results for *Brachypodium pinnatum* cover

| **Source** | **df** | **Mean square** | **F** | **p** |
| --- | --- | --- | --- | --- |
| Land use | 1 | 0.297 | 2.65 | 0.245 |
| Site | 2 | 0.215 | 1.91 | 0.343 |
| Land use × Site | 2 | 0.112 | 3.14 | 0.053 |
| Sampling error | 46 | 0.036 | - | - |

**2B)** SAS 9.2 code and table of the results for the ANCOVA for testing mean differences in *Brachypodium pinnatum* cover between different types of land use regimes, having Elevation and Slope as a covariates.

*#MODEL: Randomized complete block design, with A as factor and R as blocks, and X1 and X2c as covariates;*

*# testing the interactions with X1 and X2c*

*PROC GLM DATA=M1;*

*CLASS A R;*

*MODEL X4= X1 X2c A R A*R X1*A*R X2c*A*R/solution SS3;*

*RUN;*

*#no significant interactions detected*

*PROC GLM DATA=M1;*

*CLASS A R;*

*MODEL X4= X1 X2c A R A*R/SS3;*

*TEST H=A R E=A*R;*

*LSMEANS A*R/STDERR TDIFF;*

*LSMEANS A R/STDERR TDIFF E=A*R;*

*RUN;*

*OUTPUT OUT=RES7 PREDICTED=PREDICT RESIDUAL=RESID;*

*PROC UNIVARIATE DATA=RES7 PLOT NORMAL;*

*VAR RESID;*

*RUN;*

*PROC ANOVA DATA=RES7;*

*CLASS A R;*

*MODEL RESID = A*R;*

*MEANS A*R/HOVTEST=BARTLETT;*

*RUN;*

ANCOVA result for *Brachypodium pinnatum* cover

| **Source** | **df** | **Mean square** | **F** | **p** |
| --- | --- | --- | --- | --- |
| Elevation | 1 | 0.002 | 0.06 | 0.803 |
| Slope | 1 | 0.033 | 0.92 | 0.344 |
| Land use | 1 | 0.201 | 1.56 | 0.338 |
| Site | 2 | 0.028 | 0.22 | 0.822 |
| Land use × Site | 2 | 0.129 | 3.53 | 0.038 |
| Sampling error | 44 | 0.037 | - | - |
